# Supplementary material for: Resegmentation is an ancestral feature of the gnathostome vertebral skeleton
Source: eLife. 2020 Feb 24;9:e51696. doi: 10.7554/eLife.51696 (PMC7064331; doi:10.7554/eLife.51696)
Supplement: Transparent reporting form [file elife-51696-transrepform.docx]

***eLife’s* transparent reporting form**

We encourage authors to provide detailed information *within their submission* to facilitate the interpretation and replication of experiments. Authors can upload supporting documentation to indicate the use of appropriate reporting guidelines for health-related research (see [EQUATOR Network](http://www.equator-network.org/%20)), life science research (see the [BioSharing Information Resource](https://biosharing.org/)), or the [ARRIVE guidelines](http://www.plosbiology.org/article/info:doi/10.1371/journal.pbio.1000412) for reporting work involving animal research. Where applicable, authors should refer to any relevant reporting standards documents in this form.

If you have any questions, please consult our Journal Policies and/or contact us: [editorial@elifesciences.org](mailto:editorial@elifesciences.org).

**Sample-size estimation**

- You should state whether an appropriate sample size was computed when the study was being designed
- You should state the statistical method of sample size computation and any required assumptions
- If no explicit power analysis was used, you should describe how you decided what sample (replicate) size (number) to use

Please outline where this information can be found within the submission (e.g., sections or figure legends), or explain why this information doesn’t apply to your submission:

The somite and vertebral counts included samples of 17 and eight individuals, respectively. We did not conduct any statistical analyses on these, except to calculate the mean. The sample sizes were decided by availability of skate embryos and hatchlings.

**Replicates**

- You should report how often each experiment was performed
- You should include a definition of biological versus technical replication
- The data obtained should be provided and sufficient information should be provided to indicate the number of independent biological and/or technical replicates
- If you encountered any outliers, you should describe how these were handled
- Criteria for exclusion/inclusion of data should be clearly stated
- High-throughput sequence data should be uploaded before submission, with a private link for reviewers provided (these are available from both GEO and ArrayExpress)

Please outline where this information can be found within the submission (e.g., sections or figure legends), or explain why this information doesn’t apply to your submission:

We had a total of 38 biological replicates (i.e., we injected two adjacent somites in each embryo, and 38 embryos survived to fixation) for the trunk fate mapping experiment. Of these, we sectioned and analyzed 34, and found dye in the vertebrae of 27/34. Of the 27 embryos with vertebral cartilage labelling, 22 showed clear tetrapod-like resegmentation, while five had aberrant dye labelling patterns.

We had 31 biological replicates for the tail fate mapping experiment. Of these, we sectioned and analyzed 24 and found dye in the vertebral cartilage of all embryos. 19/24 individuals showed clear evidence for the resegmentation pattern described in the text, while four showed aberrant dye labelling patterns. One embryo was excluded because the injection site was so far posterior as to not have differentiated vertebrae.

We had 17 replicates for the cloaca fate mapping experiment. Of these, we analyzed nine individuals and excluded none.

Individuals were excluded from the reported total only if no dye was present in the vertebral cartilage (indicating that dye was not successfully incorporated into somite cells, or that injection placement was too dorsal in the somite).

We performed each *in situ* hybridization experiment twice, on tissue from different embryos.

Replicate information can be found in the results section.

**Statistical reporting**

- Statistical analysis methods should be described and justified
- Raw data should be presented in figures whenever informative to do so (typically when N per group is less than 10)
- For each experiment, you should identify the statistical tests used, exact values of N, definitions of center, methods of multiple test correction, and dispersion and precision measures (e.g., mean, median, SD, SEM, confidence intervals; and, for the major substantive results, a measure of effect size (e.g., Pearson's r, Cohen's d)
- Report exact p-values wherever possible alongside the summary statistics and 95% confidence intervals. These should be reported for all key questions and not only when the p-value is less than 0.05.

Please outline where this information can be found within the submission (e.g., sections or figure legends), or explain why this information doesn’t apply to your submission:

This information does not apply to our submission because we did not do any statistical analysis, aside from reporting means for vertebral and somite counts.

(For large datasets, or papers with a very large number of statistical tests, you may upload a single table file with tests, Ns, etc., with reference to sections in the manuscript.)

**Group allocation**

- Indicate how samples were allocated into experimental groups (in the case of clinical studies, please specify allocation to treatment method); if randomization was used, please also state if restricted randomization was applied
- Indicate if masking was used during group allocation, data collection and/or data analysis

Please outline where this information can be found within the submission (e.g., sections or figure legends), or explain why this information doesn’t apply to your submission:

The experiments in this study (fate mapping, in situ hybridizations) were not applicable for control and treatment groups.

**Additional data files (“source data”)**

- We encourage you to upload relevant additional data files, such as numerical data that are represented as a graph in a figure, or as a summary table
- Where provided, these should be in the most useful format, and they can be uploaded as “Source data” files linked to a main figure or table
- Include model definition files including the full list of parameters used
- Include code used for data analysis (e.g., R, MatLab)
- Avoid stating that data files are “available upon request”

Please indicate the figures or tables for which source data files have been provided:

We have uploaded .csv files of somite and vertebral count tables as source data files.
